# Puerperal Insanity

**Published:** 1899-12-23

**Authors:** F. St. John Bullen


### Hospital Clinics and Medical Progress.

### PUERPERAL INSANITY.

By F. St. John Bullen, M.R.C.S.

(*Continued from page* 176.)

**Physical Causes.**—The prolonged struggle against the altered conditions of secretion and elimination in the pregnant woman is succeeded by the physical strain of parturition, with its pains, anxiety, subsequent exhaustion, transference of nerve energy, &c., and which may be accentuated by such complications as prolonged labour, instrumental interference, hæmorrhage, multiple births. Thus, in the 160 cases of puerperal insanity in which the character of the labour was known, 27·5 per cent. were preceded by one or other of these complications.

Lastly, comes the influence of septic infection. What part this plays in the production of real insanity of the post-parturient period is yet undecided, and there are sufficient reasons for this. In the first place, it may be difficult to determine whether the condition is that of a true psychosis accompanying, perhaps dependent upon, septicæmia, or that of fever in a puerperal woman with delirium. It must be remembered that many local and subjective symptoms are masked in the insane; that disturbances of lochial and lacteal flow may be the result of nervous disturbance alone; again, that a septic origin may be unsuspected, or it may be wrongly inferred when local symptoms of uterine mischief exist, and that delirium may be greatly modified by heredity, and finally pass into a genuine insanity. The statistics as to the rôle of septic infection in the causation of puerperal insanity will vary according to the views of different writers. Thus, it is returned as an effective cause in from 20 per cent. to 70 per cent. of cases. The higher percentage can only be accounted for by the supposition that many of the cases were those of puerperal fever with active delirium, the authors including zymotic maladies and tuberculosis in the causes of the septicæmia. The smaller percentage is an average from the returns of some leading British authorities, and is compiled from cases of septicæmia not of zymotic origin with genuine insanity or acute delirious disorder which could be regarded as the main feature of the malady.

There is still another condition to be regarded in this place, namely, endogenetic toxæmia, which may determine a psychosis early after childbirth, though more probably after an extended interval. This is dependent upon the checking by some physical or emotional cause of the active depurative processes attendant upon disintegration and removal of waste tissue.

It will thus be seen that the etiology of the puerperal psychosis presents only variations of the few active causes of insanity in general. There is the almost necessary factor of hereditary predisposition, the lengthy stress of the latter part of pregnancy, the acute strain of labour with, perhaps, abnormal difficulties and sequelæ, the period of involution processes, and in addition the risk peculiar to this malady of septic infection. The varieties of the psychosis may be understood partly from the nature, combination, and potency of the predisposing and exciting causes. According to the impressionability of the subject and the strength of the exciting circumstances, the mental disturbance at birth may vary from hysterical excitement to a frenzy in which the mother may endanger her own life or that of her offspring, or a mild delirious state lasting from a few hours to a day or two may be met with. Corresponding to the establishment of the milk flow, again, there may be some mental disturbance, usually of delirious type and lasting from the second to fourth day; it may, however, pass, with or without remission of symptoms into definite insanity. This derangement is either dependent on the nervous excitement attending the establishment of the lacteal flow, or to local mammary condition, a slight pyrexia, or to a mild septicæmia; any of which may act on a sufficiently unstable brain. During this delirious state the danger of infanticidal impulse is considerable and should never be disregarded.

Puerperal insanity proper, like other forms of the psychoses, shows variations according to the intrinsic and extrinsic causes producing it, and though usually coloured by the special nature of the latter, is not necessarily so. Its onset has already been spoken of, and here it may be said that those inferences which apply to insanity as a whole have a similar application in this disorder. The slower the development of the psychosis after childbirth, the more delayed the treatment and the less sthenic the type, the more unfavourable is the outlook in regard to the time and eventuality of recovery. States of excitement, classed as mania, are the most frequent; calculated on a large number of cases they are to conditions of depression as 2½ to 1. They appear earlier, too, than the latter, the onset of which is not seldom postponed to a fortnight after delivery, and they pursue a shorter course. Both the maniacal and melancholic form supervene on exhaustion, but the first more generally follows on acute and the second on gradual asthenia, not infrequently being associated with phthisis and anæmia.

The maniacal class includes a very large percentage of acute delirious forms, more than half, in some returns; the cases of melancholic type are, in the main, more mild in character. There are some clinical features which pertain to puerperal insanity, as a pyschosis with sudden and deep reductions in consciousness, and frequently a marked sexual element. In the first place is observable a particularly high ratio of impulsive tendencies, and in the second, salacity, religiosity, and stupor. Be it understood these are by no means concomitants of all forms of puerperal insanity, but one or other is often present in typical examples of this. As with so many cases of rapid mental reductions, the state is one of profound apprehension, and often issues in sudden action of homicidal or suicidal kind. It will be found that either impulse is probably based on a condition of intense fear, from some terrifying delusion; especially is this the case in infanticidal attempts. The suicidal tendency is thus found in the maniacal as well as melancholic states, and is indeed present, more or less actively, in at least 35 per cent. of all cases. There is often evidence of a salacious train of thought shown indirectly by jealous accusations of husband, of nurses, &c., in a direct way by indecent behaviour and depraved habits. These are probably the result of some peripheral genital irritation. In the exalted forms of mania, as is common, an obtrusive religiosity is associated with the preceding and also at one or other period in the course of the malady there is stupor in some degree. In quite a quarter of the cases this is present, and as in all forms of stupor where a delusion may be presumed still active, the risk of impulsive acts needs bearing in mind.

Beyond these points there is nothing peculiar, as a rule, in the symptoms of puerperal insanity. It only needs to be mentioned that, with or without an acute period, chronic systematised insanity may develop, but without any frequency justifying special association with the puerperium, and again that a morbid mental and moral state short of actual insanity may be left after the apparent recovery from acute symptoms. Hallucinations occur with moderate frequency, the aural being most prominent; delusions occur in large proportion, and are usually of a painful character.

